# Somatosensory Deficits After Stroke: Insights From MRI Studies

**DOI:** 10.3389/fneur.2022.891283

**Published:** 2022-07-12

**Authors:** Qiuyi Lv, Junning Zhang, Yuxing Pan, Xiaodong Liu, Linqing Miao, Jing Peng, Lei Song, Yihuai Zou, Xing Chen

**Affiliations:** ^1^Department of Neurology and Stroke Center, Dongzhimen Hospital, The First Affiliated Hospital of Beijing University of Chinese Medicine, Beijing, China; ^2^Department of Integrative Oncology, China-Japan Friendship Hospital, Beijing, China; ^3^Institute of Neuroscience, Chinese Academy of Science, Shanghai, China; ^4^School of Acupuncture-Moxibustion and Tuina, Beijing University of Chinese Medicine, Beijing, China; ^5^Beijing Institute of Technology, Beijing, China

**Keywords:** stroke, somatosensory system, somatosensory deficits, MRI, neural plasticity

## Abstract

Somatosensory deficits after stroke are a major health problem, which can impair patients' health status and quality of life. With the developments in human brain mapping techniques, particularly magnetic resonance imaging (MRI), many studies have applied those techniques to unravel neural substrates linked to apoplexy sequelae. Multi-parametric MRI is a vital method for the measurement of stroke and has been applied to diagnose stroke severity, predict outcome and visualize changes in activation patterns during stroke recovery. However, relatively little is known about the somatosensory deficits after stroke and their recovery. This review aims to highlight the utility and importance of MRI techniques in the field of somatosensory deficits and synthesizes corresponding articles to elucidate the mechanisms underlying the occurrence and recovery of somatosensory symptoms. Here, we start by reviewing the anatomic and functional features of the somatosensory system. And then, we provide a discussion of MRI techniques and analysis methods. Meanwhile, we present the application of those techniques and methods in clinical studies, focusing on recent research advances and the potential for clinical translation. Finally, we identify some limitations and open questions of current imaging studies that need to be addressed in future research.

## Introduction

Stroke is the leading cause of death, adult disability, and neurological sequelae worldwide. Stroke survivors often present diverse neurological deficits, such as hemiparesis, spasticity, aphasia, cognitive impairment, and somatosensory deficits. Somatosensory deficits are a class of common and frequent symptoms, with a reported prevalence ranging from 50 to 80% of stroke survivors (1). Stroke patients exhibit varying degrees of somatosensory deficits. It can be characterized as an absence, decrease, increase, or distortion relative to normal sensory sensation. Post-stroke somatosensory deficits are due to lesions in the central nervous systems and often combine with other functional disturbances, particularly motor weakness. The impairment of the somatosensory system may further aggravate the motor function because somatosensory feedback is necessary for the execution of movement (2–5). Previous studies have shown that somatosensory performances are strong predictors of treatment-induced functional gains (6, 7). Thus, baseline somatosensory integrity is important for perception and action. Also, there is evidence that pure sensory deficits are reported in ~10% of acute stroke patients (8). It might affect almost all areas of daily life, including mood, mental stamina, and working performance, even in patients with good motor function. Although somatosensory deficits are a major complaint in a large number of stroke patients, there are only scarce data on the open question that which brain regions are causally involved in the occurrence and recovery of post-stroke somatosensory deficits.

Nowadays, the rise of imaging techniques has greatly improved our understanding of the anatomy and function of brain regions related to the processing of somatosensory information. Particularly, magnetic resonance imaging (MRI) is well-suited to unravel neural substrates linked to functional disorders, owing in part to its high spatial accuracy and safety. In the past decades, the initial analyses of somatosensory deficits by using MRI were descriptive. Previous studies have proved that lesions in multitude brain areas, such as the thalamus, parietal, medial lemniscus, pons, corona radiata, etc. were associated with somatosensory deficits (9–12). However, these results were only descriptive but not relied on statistics. It is urgent to elucidate to what extent lesions in brain regions affect sensory modalities and which activation patterns are associated with somatosensory function. Advances in MRI techniques are making this a reality. As for morphological signs, cortical thickness mapping and voxel-based morphology are two advanced methods to detect subtle structural changes (13, 14). Because of this, many studies have focused on the mapping of symptoms to a focal lesion. In addition, the evolution of functional MRI (fMRI) to permit the investigation of functional segregation and integration is an inspiring development to better understand the activation of specific brain regions and the communication between distributed areas (15, 16). The applications of interdisciplinary approaches in the fMRI data analysis allow the detection of function changes at the group level, and even to make individual-level predictions. The evidence of changes after stroke using certain MRI modalities is increased and could provide insights into the morphological and functional signs of post-stroke deficits. Furthermore, a comprehensive understanding of neural mechanisms could provide personalized therapy to stroke patients. Thus, more detailed analyses of different somatosensory modalities need to be conducted *via* innovative MRI studies. To address current gaps in knowledge, this review summarizes progress in the clinical application of MRI in patients with post-stroke somatosensory deficits, highlighting new insights from these studies.

## Methods

A literature search in the electronic medical databases PubMed, Web of Science, and Google Scholar from 1980 to October 2021 was undertaken by two independent researchers. Key search terms were: (stroke OR Post-stroke OR Cerebrovascular Accident OR Cerebrovascular Apoplexy OR Apoplexy OR Brain Vascular Accident OR Cerebrovasc* OR brain* OR brain vasc* OR hemipleg* OR apoplex* OR CVA OR TIA) AND (somatosensory deficits OR somatosensory impairment OR sensory impairment OR sensory stroke) AND (magnetic resonance imaging OR MRI OR structural MRI OR voxel-wise OR voxel-based morphometry OR VBM OR functional MRI OR fMRI OR ReHo OR ALFF OR functional connectivity OR neuroimaging). The languages of the articles were restricted to English, and the search strategy for each database was based on its unique characteristics. The inclusion criteria were (1) involving stroke patients (ischemic or hemorrhagic) as defined by the World Health Organization; (2) presence of somatosensory deficits; (3) using at least one of the MRI modalities. The exclusion criteria were (1) case report or case serial; (2) without any somatosensory examinations. Data should be extracted from the included studies into a standard form concerning the name of the first author, publishing year, sample size, participants' condition, lesion locations, clinical scales, fMRI method, and main findings. Two researchers independently carried out data extraction based on a pre-defined form and any disagreements were resolved by the third research.

## Results

**Figure 1:**
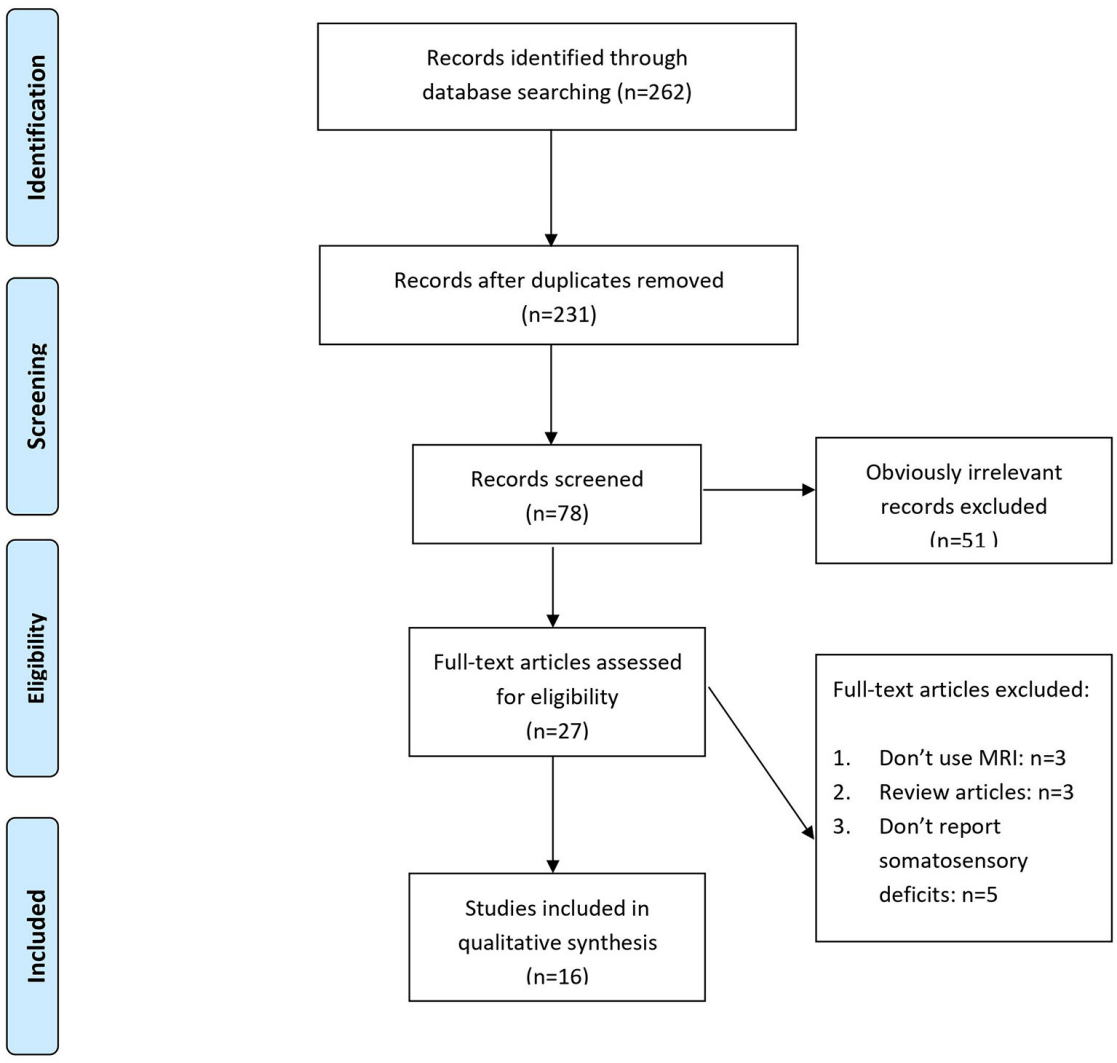
Flow chart for study selection.

The database search yielded 262 articles. 31 duplicates were removed, and 153 articles were excluded after screening titles and abstracted. Of the 27 potentially relevant reports, 16 studies proved eligible after full-text screening (Figure 1). Suitable studies included in this review were extracted in Table 1. We will focus on the MRI findings in post-stroke somatosensory deficits while reviewing the plasticity of the somatosensory map. The comparisons of different MRI techniques are also within the scope of this paper. Furthermore, a brief overview of the somatosensory system will be described in the following section for easy understanding.

**Table 1:** Overview of studies reporting MRI findings in stroke patients with somatosensory deficits.

**Reference**	**Participant**				**Outcome measures**		
	**Subjects**	**Condition**	**Lesion location**	**Diagnosis/scales**	**Imaging modality**	**Analytical method**	**Main findings**
Baier et al. (17)	24IS	Acute	Unilateral IC	QST	sMRI	VLSM	Lesions of the posterior IC may cause deficits in temperature perception.
Preusser et al. (18)	61patients	Mainly chronic IS	Not in the postcentral gyrus and ascending afferent pathway	Touch impairments examination	sMRI	VLSM	Ventral pathway of somatosensory perception links to the perception of touch.
Meyer et al. (19)	38IS or HS	Acute	Across the entire hemisphere	NIHSS, Em-NSA, PTT, SSEP	sMRI	VLSM	Stroke lesions in the superior thalamocortical radiation associate with exteroceptive and proprioceptive deficits in the arm and hand. The lesion patterns accounted for touch perception were also involved in the perception of pressure, pinprick, and proprioception.
kessner et al. (20)	101IS	Acute	Predominantly in the MCA territory	RASP, NIHSS	sMRI	VLSM	Significant associations between somatosensory deficits with lesions in SI, SII and insular were found in acute phase but not in chronic phase.
Kessner et al. (21)	101IS	Acute	Across the forebrain	RASP, NIHSS	sMRI	Structural brain connectome	Lesion volume was associated with somatosensory deficits and white matter network disruption was stronger predictor than gray matter damage.
Chen et al. (22)	8SP/15HC	Acute	In or inferior to the thalamus	Reported limb numbness	fMRI	ReHo	ReHo in the left middle temporal gyrus decreased in the patients.
Dinomais et al. (23)	14SP	Chronic	6 MCA/8 median periventricular	2-point discrimination	fMRI	seed-based	Reduction of FC within the somatosensory network were associated with sensory deficits.
Goodin et al. (24)	28SP/14HC	Chronic	Spread over frontal, parietal, temporal cortical regions; and within thalamic and striatum regions	TDT, WEST	fMRI	Seed-based	Stroke groups showed qualitative disruption of FC between the S1, S2 and inter-hemispheric regions including the cerebellum.
He et al. (25)	31TI/31HC	Chronic	In the thalamus	FLA	fMRI	ICA	Lesioned thalamus could lead to increased FC between the SMN and perilesional thalamus.
Chen et al. (26)	31TI/32HC	Chronic	In the thalamus	FLA	s-&fMRI	Multimodal MRI analyses	Thalamic lesions resulted in increased functional coupling to the non-atrophic S1 region.
Chen et al. (22)	8SP/15HC	Acute	In or inferior to the thalamus	Reported limb numbness	fMRI	seed-based	FC between the stroke locations and the left middle temporal gyrus was increased in stroke patients compared with HC.
Chen et al. (22)	8SP/15HC	Acute	In or inferior to the thalamus	Reported limb numbness	fMRI	Graph theory	Specific stroke lesions might cause local changes but not changes in the whole-brain functional network.
Lee et al. (27)	11HS	Chronic	In the thalamus	NSA	fMRI	Seed-based	The proprioceptive function of the affected hand appears to have recovered mainly *via* the primary SMN.
Carey et al. (28)	19SP/12HC	Subacute	11 subcortical lesions/ 8 cortical lesions	WPST, fTORT, temperature examination, TDT	fMRI	Random effects analysis	There was no significant difference in touch impairment between stroke subgroups. The patterns of correlated brain activity associated with touch discrimination in two subgroups were different.
Carey et al. (29)	11SP	Chronic	4 subcortical lesions/ 7 cortical lesions	WEST, WPST, temperature examination, TDT	fMRI	Random effects analysis	Patients with subcortical lesions activated the ipsilesional supramarginal gyrus and no common activation in stroke patients with subcortical lesions
Liang et al. (30)	40SP	Chronic	Not reported	TDT	fMRI	LOFC&HOFC+ML	A robust feature selection approach combined with machine learning could provide a possible avenue for linking stroke impairment to functional brain networks.
Yassi et al. (31)	15IS/7HC	Acute	Mainly in the subcortical regions	NIHSS	s-&fMRI	VBM&ICC	The thalamus would be a site of structural and functional change even when the stroke lesion is in a remote location.
Bannister et al. (32)	10SP/10HC	Chronic	Half in the thalamus, half in the SI and/or SII	TDT, WPST, fTORT, temerature examination	fMRI	Seed-based	The disrupted interhemispheric FC for the SI seed was observed at 1-month post-stroke and some return of interhemispheric FC can be seen at 6 months.

*ALFF, amplitude of low frequency fluctuations; CTR, central thalamic radiation; Em-NSA, Erasmus MC modification of the (revised) Nottingham sensory assessment; FC, functional connectivity; FLA, fugl-Meyer and Lindmark assessment; fMRI: functional MRI; fTORT, functional tactile object recognition test; HC, health control; HOFC, high-order functional connectivity; HS, hemorrhagic stroke; ICA, independent component analysis; IC, insular cortex; IS, ischemic stroke; ICC, intrinsic connectivity contrast; LOFC, low-order functional connectivity; MCA, middle cerebral artery; ML, machine learning; MRI, magnetic resonance imaging; NIHSS, National Institutes of Health Stroke Scale; PTT, perceptual threshold of touch; QST, quantitative sensory testing; RASP, rivermead assessment of somatosensory performance; ReHo, regional homogeneity; SI, primary somatosensory cortex; SII, secondary somatosensory cortex; sMRI, structural MRI; SP, stroke patients; SSEP, somatosensory evoked potentials; TDT, tactile discrimination test; TI, thalamic infarction; TFPT, tactile form perception test; VBM, voxel based morphometry; VLSM, voxel-based lesion-symptom mapping; WEST, Weinstein enhanced sensory test; WPST, wrist position sense test*.

### An Overview of the Somatosensory Pathway

Knowledge of the layout and wiring of the somatosensory system is important to understand the post-stroke somatosensory deficits. The purpose of this section is to provide a comprehensive overview of somatosensory deficits, along with a brief overview of the anatomical and functional features of the somatosensory system.

The somatosensory system has two functions: somatosensory perception and guidance of action. The former means conscious perception and recognition, including exteroceptive and proprioceptive sensation (33). Exteroception sensation is the ability to recognize the superficial skin information from mechanoreceptors, thermoreceptors, and nociceptors, whereas proprioception sensation includes deep sensory input from muscle and joint receptors. Beyond perception, somatosensory processing also has the function of the guidance of action (34). Under normal conditions, the perception and action should act in a coordinated fashion to maintain the conduction of sensory information. Those functional dissociations suggest that separate neural pathways are involved in the processing of somatosensory input for conscious perception and the guidance of action.

Most information acquired from peripheral sensory receptors (except face) will be sent to the spinal cord, while unconscious proprioceptive information will be directly sent to the cerebellum through the spinocerebellar tract. From the spinal cord, the information will then ascend to the ventral posterior lateral (VPL) of the thalamus *via* the medial lemniscus or spinothalamic tract. The thalamus was long described as a relay station for sensory information transmitted from the periphery to the cortex, and it plays a critical role in nearly all aspects of cortical information processing, including carrying the information to the cortex and modulating the cortical processing (35–37). Thus, even a small thalamic lesion can cause deafferentation and a loss of somatosensory information (38, 39). The somatosensory areas of parietal cortex contain multiple topographic maps of the body surface, famously depicted as the “homunculus” of Penfield (40). Lesions of the parietal lobe are the most frequent cause of somatosensory deficits (35). Axons originating in the VPL will convey information mostly into the anterior parietal cortex (APC), which is viewed as the anatomical site of the primary somatosensory cortex (SI). It consists of four different areas (Brodmann areas 3a, 3b, 1, and 2) and each area contains a separate representation of the contralateral body (41).

Moreover, the secondary somatosensory cortex (SII), the posterior parietal cortex (PPC), and the insula are other key areas in the cortical somatosensory network and are thought to be involved in higher-order sensory processing. The SII is found in the parietal operculum and plays a role in information integration. It will receive the input mainly transmitted from the SI and directly receive some input from the thalamus (42, 43). The insula is located on the deep side of the Sylvian fissure and has many connections with multiple different brain regions. It plays an important role in the integration of somatosensory information with other sensory modalities, particularly the control of autonomic systems and pain encoding (44–47). The PPC is important for spatial processing and visually guided action, and the lesions of the PPC could lead to hemispatial neglect, with which patients will be unaware of sensory deficits (48, 49). Notably, evidence indicates the existence of at least two different streams in the processing of non-painful somatosensory stimuli. One stream projecting from the APC *via* the SII to the posterior insula is considered the ventral pathway and subserves perceptual recognition and learning (50–52). Another pathway terminating in the PPC is considered the dorsal pathway and subserves both perception and action (34). Both streams subserve the recognition and perception of somatosensory information, whereas action-related processing mainly occurs in the PPC, which can be assumed that the PPC could accumulate sensory input to encode action plans (53, 54). Besides, somatosensory processing also has a connection with the cerebellum (55–58). The cerebellum is associated with the somatosensory cortex afferently and efferently (59). It is responsible for monitoring sensory information and is also involved in forming predictive associations between sensory inputs and motor outputs (60, 61).

### Structural MRI Findings in Post-stroke Somatosensory Deficits

#### Voxel-Based Lesion-Symptom Mapping (VLSM)

Given that the lesion site is a critical source of heterogeneity associated with sensory stroke, innovative methods have been developed to investigate the contribution of lesion location to sensation outcome. Voxel-based lesion-symptom mapping (VLSM) is one of the most common methods to estimate the statistical contribution of single damaged voxels to a certain sensation symptom, by normalizing the structural MRI images to standard space, using the non-parametric mapping toolbox (NPM) implemented in MRIcron to do a VLSM analysis and applying an appropriate algorithm (e.g., Liebermeister Test) to assess between-group effect (62, 63). Baier et al. (17) first used this approach to explore the statistical-based association between lesion locations and somatosensory deficits in stroke patients. They recruited 24 acute stroke patients with lesions affecting the unilateral insular cortex (IC) and did a voxel-wise analysis of lesion location using some Quantitative sensory testing (QST) parameters as binary measurement variables. The results showed that stroke lesions affecting parts of the posterior IC are significantly associated with increased cold and warm detection thresholds, which is consistent with the notion that the posterior IC may be a critical brain region responsible for innocuous temperature perception (44, 45). Furthermore, they didn't find a significant association between the IC and pain thresholds, whereas some previous case studies and functional imaging studies have revealed that lesions of IC could also lead to pathological pain (12, 64–66). This discrepancy may be since the patients in this study mainly had stroke lesions in the posterior IC, which is more involved in the processing of non-nociceptive stimuli rather than nociceptive stimuli (67, 68). With respect to light touch perception, there has been emerging evidence that it causally involves the SI (69, 70). To investigate which brain areas downstream of the SI are specifically responsible for touch perception, Preusser et al. (18) identified 61 patients (mainly suffered from a chronic ischemic stroke) without lesions in the SI and then compared patients with impaired touch perception (i.e., hypoesthesia) to patients without such impairments. They found that many brain regions, including the SII (predominately anterior subdivisions), as well as the anterior and posterior IC, putamen, together with the white matter fiber bundles reaching to the prefrontal cortex, contribute to the perception of touch. Hence, to some extent, these findings also confirmed previous speculations on a perception-related ventral pathway originating in the SI, passing the SII before ending in the IC as we mentioned in the previous section (34). This ventral pathway might be particularly associated with material object properties (e.g., texture), which are the province of touch (71, 72).

Considering that the abovementioned two studies provided information only from the perspective of temperature or touch perception, Meyer et al. (19) carried out a similar study in acute stroke patients to investigate which regions are implicated in different exteroceptive and proprioceptive somatosensory deficits by using four different clinical scales. The results showed that two core brain regions, including the parietal white matter and the SII close to the IC (the insulo-opercular cortex), could be the most vulnerable brain regions to cause impaired touch perception, which are in concordance with the findings from Preusser et al. (18). Similarly, they found that these lesion patterns were also involved in the perception of pressure, pinprick, and proprioception, despite the subtle differences. From this, it can be assumed that different somatosensory modalities may be affected by these same lesioned brain areas. This hypothesis was partially supported by the observations provided by Kessner et al., (20) who examined a sample of 101 stroke survivors by using VLSM and the subitems of the Rivermead Assessment of Somatosensory Performance (RASP). The RASP has seven different subitems, which can assess both simple perceptions (pressure and light touch) and more integrative tasks (2-point discrimination, sensory extinction, joint position, and movement sense). They found in the acute phase of stroke patients, SI and SII showed up for virtually all somatosensory performance. Besides, they also observed that different somatosensory modalities represented different lesion patterns of brain regions, for example, the putamen contributed to proprioception and sensory extinction; frontal-subcortical areas close to the frontal insular cortex and the external capsule were associated with proprioception, sensory extinction, pressure, and light touch.

#### Structural Brain Connectome

While these VLSM studies focus on the localized relationships between lesion patterns and behavior, lesion-network studies of the structural connectome could also reveal the neuroanatomical correlates of post-stroke deficits (73, 74). The structural brain connectome is a novel approach in clinical neuroscience. It could be seen as a network, including a finite set of nodes and edges, which represents the interconnections between different gray and white matter areas. Kessner et al. (21) first conducted a structural MRI study to investigate the correlation between stroke lesions on structural connectome and post-stroke somatosensory deficits. They recruited 101 acute ischaemic stroke patients with somatosensory deficits, which were evaluated by RASP. They used two distinct methodologies, Node Destruction (NoDe) and Change in Connectivity (ChaCo), respectively, to evaluate the effect of stroke lesion on nodes and edges of the structural connectome. The results showed that the impaired sensation may result from stroke lesions in the anterior parietal and superior temporal lobes, and the extent of the acute infarct was strongly associated with somatosensory deficits, which is in accordance with the conclusion that lesion volume could explain the severity of clinical symptoms (75). Besides that, the results also suggested that disruption of white matter pathways to the identified regions (mainly in the supramarginal and transverse temporal gyri) accounts for a larger proportion of variation in the sensation outcomes, which indicates that white matter integrity is important for normal processing of somatosensory inputs. To summarize, the VLSM studies have revealed that stroke lesions in the SI, SII, and IC are associated with somatosensory symptoms, but exactly which lesion patterns of brain regions lead to a certain somatosensory modality remains elusive. The structural brain connectome study provided the differential evaluation of gray and white matter disruption as structural correlates of somatosensory deficits after stroke.

### Functional MRI Findings in Post-stroke Somatosensory Deficits

A limitation of structure MRI is that it might not necessarily reflect the full function of a certain brain area. This limitation has spurred interest in the use of fMRI, as a method to elucidate the nature of brain activity in relation to behavioral outcomes. Since its emergence in the early nineties, fMRI has become one of the most commonly used neuroimaging tools to map human brain functions (76). It could reflect the change of blood oxygenation in brain regions by detecting the blood-oxygen-level-dependent (BOLD) contrast. BOLD contrast forms the basis of fMRI formation, which can be divided into task-based and resting-state fMRI(rs-fMRI) (77). In the past two decades, task-based fMRI has been used widely to identify abnormal brain function and uncover brain substrates. However, task-based fMRI of stroke studies often suffers from a large variability because it is hard for stroke patients to perform the tasks well (78). Comparatively, rs-fMRI could recognize the spontaneous activities of the brain beyond instantaneous task-induced change, which is more suitable for stroke patients. Thus, in this section, the spotlight is mainly on the rs-fMRI studies. There are two major categories of rs-fMRI analytical methods: functional segregation and functional integration. The former focuses on the specific brain region activity, including Amplitude of Low Frequency Fluctuations (ALFF) and Regional Homogeneity (ReHo). Conversely, the measurement of functional integration can observe the brain as an intercomponent network, which relies on the analysis of different brain areas in connectivity (16).

#### ALFF and ReHo Analysis

Amplitude of low frequency fluctuations and ReHo are two important approaches to describe the regional spontaneous neural activities, which were originally adopted by Zang et al. (79, 80). ALFF depicts the fluctuation amplitude of a particular voxel, measuring BOLD signals within the low-frequency band of 0.01–0.08 Hz. ReHo reflects the neural synchronization of the time course between a particular voxel with its neighboring voxels, which is sensitive to detect abnormalities. Both ALFF and ReHo depend on similar neurovascular components and those two parameters usually have a positive correlation. In most cases, higher synchronicity within a voxel (increased ReHo values) can cause higher fluctuations (increased ALFF values), and in turn, lead to increased synchronicity across voxels. A previous study on healthy subjects has revealed that higher BOLD amplitudes and synchronicity of the SI hand region at resting-state, as measures of ALFF and ReHo, respectively, are associated with better tactile discrimination abilities of the contralateral hand (81). This finding is consistent with previous studies indicating that the localized BOLD activity can indeed be considered a surrogate marker of symptoms and behaviors (82, 83). Therefore, it is reasonable to believe that the baseline activity in the sensory-related regions assessed by local ALFF and ReHo values could be related to sensory performance. Based on this, Zhu et al. (84) utilized both the ALFF and ReHo to compare 19 stroke patients with 15 healthy controls (HC) in three different frequency domains. The between-group differences were found predominately occurring in the parietal cortex, which is in accordance with previous studies showing abnormalities in the parietal cortex in stroke patients (85–87). These results are plausible, as the parietal cortex is well-known to be involved in somatosensory processing. Thus, there is reason to believe that the vulnerability of the parietal cortex could be an explanation for the abnormal sensory after stroke. Moreover, Chen et al. (22) recruited eight acute ischemic stroke patients suffering from limb numbness with lesioned areas in or inferior to the thalamus and then compared those patients with 19 healthy controls by using ReHo. The significantly lower ReHo values in the contralateral middle temporal gyrus/superior occipital gyrus have been reported in stroke patients compared to healthy controls. Considering that those regions are proven to be important in the integration of various sensory information, it can be assumed that the decreased activity of the temporal area induced by impaired information input from the thalamus might cause abnormal somatosensory processing (88, 89). Those findings together converge to demonstrate the applicability of analyzing ALFF and ReHo in stroke patients and regional properties can provide a comprehensive understanding of the neural pathology of post-stroke somatosensory deficits. Moreover, those results also indicate a positive correlation between somatosensory functions and cortical activation in stroke patients and also demonstrate the applicability of analyzing ReHo or ALFF in stroke patients with neurological deficits (90).

#### Functional Connectivity Analysis

Functional connectivity (FC) is a relatively new topic of research that measures the connectivity among different brain regions and could reveal how brain networks dynamically change during diseases. This method can predict up to 80% of brain structural connections (91, 92). Nowadays, many different analytical methods have been introduced to study FC of rs-fMRI, including Seed-Based, independent components analysis (ICA), and graph theory (77). Seed-Based analysis was the first method to analyze the resting-state network, which was proposed by Biswal et al. (93). It is a hypothesis-driven approach that requires the priori seed or region of interest (ROI) selection at first and then correlates those predefined seeds with other regions activated at the same time. The seed-based analysis relies on the prior knowledge or experience and to some extent, neglects other potential changes beyond seeds. Different from seed-based analysis, ICA is a data decomposition-based method, which detects extensive connectivity networks without seed selection. Based on the independence and non-Gaussianity of the signal, it could utilize mathematical algorithms to decompose the signal from the whole brain to spatially and temporally independent components (77, 94, 95). Finally, the application of graph theory in brain FC analysis can establish models of complex network functions with spatial details. It is a model-free connection technique and makes a distinction between nodes and edges of a network (96). It can provide information about both the integration and segregation of brain networks by using global and local graph theory metrics (97, 98). In general, the rs-fMRI connectivity has been considered a promising approach to offer an excellent opportunity to explore neural substrates and dysfunctional mechanisms *in vivo* after stroke. In addition, there are various software, such as SPM, DPARSF, REST, MECODIC tool of FSL, CONN etc., for pre-processing and processing fMRI data.

The high degrees of inter-hemispheric symmetry evaluated by FC analysis is one of the notable features of the ahealthy brain. Accumulating evidence has proven a reduction of inter-hemispheric connectivity between homotopic cortical areas after suffering from stroke (99–101). Dinomais et al. (23) proposed the hypothesis that stroke patients with sensory deficits might have less FC in their somatosensory cortical networks. They investigated the somatosensory network by using seed-based analysis in two groups children with middle cerebral artery (MCA) strokes or periventricular lesions, respectively. The results showed that patients with chronic MCA strokes, who had higher impairments in 2-point discrimination, represented significant reductions of FC within the lesioned somatosensory cortex and the SII only. Furthermore, the unexpected lateralization found in SII rather than SI led to a hypothesis that a more pronounced affection for SII may indicate a stronger sensory deficit. Similarly, Goodin et al. (24) examined FC in 28 stroke survivors with tactile impairments and 14 age- and sex-matched HC using seed-based analysis. Inter-hemispheric connectivity was reported to be greater in HC compared to the stroke cohort who had impairments in touch sensation assessed by the Tactile Discrimination Test (TDT). Those findings of less inter-hemispheric connectivity in the stroke patients with somatosensory deficits are in line with the current study that reducing hemispheric FC can reflect corresponding neurological deficits (102).

The study conducted by He et al. (25) sought to determine whether lesioned thalamus could lead to FC alterations in the somatosensory-related regions. In total, 31 chronic thalamic infarction patients presented with contralesional somatosensory deficits, and 31 HC underwent rs-fMRI and neurological assessments. After comparing the data by using ICA, decreased FC was observed in the ipsilesional SI, revealing that less activation in the SI induced by subcortical infarction (i.e., thalamic infarction) is associated with somatosensory deficits (28). In addition, they also reported increased FC in the perilesional areas of the affected thalamus. This finding could be due to the reorganization of the thalamus, which may appear in the session of recovery of thalamic infarction patients (27). Similarly, Chen et al. (26) reported that in 31 patients with chronic thalamic infarction, the region (the ipsilesional middle SI) exhibiting increased FC with the thalamus was adjacent to the region (the ipsilesional top SI) exhibiting decreased cortical volume. The secondary impairment in the top SI may be implicated in somatosensory deficits, while the increased FC in the non-atrophic SI region might be related to axons sprouting to establish new projection patterns and connections (103, 104). Another seed-based study using the lesion-induced method also showed that in eight acute thalamic stroke patients presenting with limb numbness, increased FC was observed between the contralateral middle temporal gyrus and the stroke areas (22). Those findings could be interpreted in terms of compensation, which is consistent with the theory that the brain could compensate for damages through reorganization and the creation of new connections among undamaged neurons (105). Alternatively, another possibility is that increased FC might reflect a primary pathophysiological change (106). Whether the increased FC within the sensory cortex may serve as a compensation effect or a pathophysiological change remains speculative and needs further evidence. Moreover, based on graph theory, previous studies demonstrated that stroke patients had significantly lower small-worldness than healthy controls (107, 108). However, Chen et al. (22) detected differences in the whole-brain functional network between healthy controls and stroke patients (stroke was located only in or inferior to the thalamus) by using graph theoretical analysis but didn't find any significant changes in the whole brain, which lead to the hypothesis that specific lesioned regions might cause specific changes in local but not influence the topological properties of the whole brain.

The FC analysis could measure the temporal correlation of activity between different brain regions, but this correlation is now considered low-order functional connectivity (LOFC). Benefiting from machine learning approaches, the concept of high-order functional connectivity (HOFC) has been purposed to depict the important interactions among all correlations captured by conventional FC analysis, which is referred to as “correlation of the correlation” (109, 110). The application of a high-order model may be more accurate in functional segmentation of the brain and useful in providing high-level information for predicting functional outcomes (111–113). Currently, various machine learning methods have been applied to analyze fMRI data on brain disorders (114–116). Commonly used machine learning algorithms in this field include logistic regression, Least Absolute Shrinkage and Selection Operator (LASSO) regression, Support Vector Machine (SVM), random forest, gradient-tree booting, fully-connected network, convolutional network, etc. (117). Liang et al. (30) conducted a preliminary study of 40 chronic stroke patients to investigate the feasibility of applying machine learning to analyze resting-state FC data in order to predict somatosensory outcomes evaluated by TDT. They used the randomized LASSO approach to construct HOFC, and then chose two machine learning models (linear regression and support vector regression) to predict somatosensory impairment from the disrupted network. The results showed that the regression model employing both LOFC and HOFC can predict TDT scores, which demonstrates that a robust feature selection approach combined with machine learning could provide a possible avenue to identify the relationship between the whole-brain connectome and stroke impairments in somatosensory function. Although it is a preliminary study, it indeed develops a possible potential to facilitate the extraction of neuroimaging biomarkers and the development of personalized treatment selection in the future.

### The Plasticity of the Somatosensory Map After Stroke

The somatosensory maps can undergo cortical reorganization when sensory input patterns change, such as altered sensory use after peripheral or central lesions (118, 119). This process can be called neuroplasticity, which refers to the capability of the nervous system to reorganize and modify the brain structure and function, yet it still locks clear definition and general theory (120, 121). From a micro-perspective, the biological phenomena, such as long-term potentiation (LTP), dendritic remodeling, and neurogenesis, are strong evidence. In a macroscopic view, the MRI and other neuroimaging techniques can also prove that the brain can be reprogrammed and structurally rebuild by revealing the activity alteration of brain regions. The process of recovery and reorganization in response to behavioral demands or injury can be well-demonstrated by several advanced MRI approaches (16). For somatosensory map plasticity, the cellular and synaptic mechanisms of use-dependent map plasticity have been best described (122, 123). However, much less is known about neural mechanisms of central lesion-induced reorganization, particularly stroke-induced neuroplasticity. Only a few longitudinal studies have explored the time course of recovery from post-stroke somatosensory deficits. Those studies suggested that patients could recover at least partially after stroke, mainly during 3–6 months, but showed strong interindividual divergence (20, 124–126). Here, we want to discuss neuronal remodeling in response to spontaneous-induced recovery of somatosensory deficits by reviewing recent MRI studies. The elucidation of the recovery mechanisms of somatosensory deficits is important to provide bases for establishing proper rehabilitation strategies (127).

As already mentioned earlier in this review, previous studies have shown that thalamic lesions could lead to increased FC within the somatosensory-related cortex, which is presumed as the manifestation of compensatory effect (22, 25, 26). For example, He et al. (25) reported on contributions of peri-lesional reorganization. They found an association of somatosensory recovery with an increase in thalamic activation in the affected hemisphere in chronic thalamic infarction patients. It can thus be speculated that the ipsilesional thalamus itself may be involved in the neuroplasticity associated with somatosensory recovery. Consistently, a previous case demonstrated that ipsilesional thalamic reorganization could occur after a thalamic stroke and it may play a vital role in recovery from such a stroke (128). Also, several studies have emphasized the important role of ipsilesional thalamic in somatosensory recovery in patients with thalamic lesions (129–131). The thalamus is the central hub of sensory function. It acts not only as a simple relay transmitting information from the periphery to the cortex but also as a high-order relay among cortical regions (132). A previous MRI study suggested that infarcts causing pure sensory were predominantly in a thalamic site (133). Thus, the integrity of ipsilesional thalamic circuitry in the recovery of somatosensory deficits after thalamic lesions appears to be important. Moreover, Chen et al. (22) attributed the sensory recovery to other unaffected somatosensory cortex or pathways. They found that in stroke patients with lesions in or inferior to the thalamus, increased FC could be observed between the lesioned locations and other remote areas, including the contralesional middle temporal gyrus and superior occipital gyrus, which could be interpreted by the concept of the cortical-thalamo-cortical loop. According to the cortical-thalamo-cortical loop theory, the thalamus is connected anatomically to the somatosensory-related cortex *via* thalamo-cortical projections (134–136). The temporal gyrus and the occipital gyrus are considered important brain regions in the thalamo-cortical circuit (89). Thus, it is reasonable to assume that the likely relevance of somatosensory recovery and alterations in peri-lesional thalamic and some remote regions might reflect the involvement of thalamo-cortical circuit in reorganization patterns induced by subcortical lesions. In addition, Lee et al. (27) also attempted to explore the underlying mechanisms of somatosensory recovery, using fMRI in 11 chronic thalamic hemorrhages. They performed fMRI by proprioceptive input and found that the recovery of proprioceptive function mainly depends on the enhancements of contralateral primary sensorimotor cortex (SM1) activation. It should be noted that proprioceptive information has a lot of effects on coordinated motor function and the degree of proprioceptive impairment can be regarded as an important predictor for motor recovery (137–140). The correlation between the improvement of somatosensory function and the activations of SM1 could lead to the conception of sensori-motor interaction. Evidence indicates that a loss of somatosensory information caused by sensori-motor disconnection could worsen motor impairments (11). Therefore, it can be assumed that the activation of SM1 during the somatosensory recovery may partly facilitate motor training effects on motor function (141, 142).

The aforementioned studies provide a general hypothesis that the subcortical-cortical circuit and the sensori-motor networks may account for somatosensory recovery after subcortical lesions. These observations have aroused scientific interest in investigating the multiple patterns of brain activation across stroke patients with different lesion locations. Evidence from the model of motor recovery showed that different patterns of activation were associated with different lesion locations (143). It, therefore, stands to reason that lesions following subcortical or cortical regions may have different activation patterns during recovery of somatosensory. Carey et al. (28) conducted a fMRI study to characterize and compare touch impairment-related brain activation between 11 stroke patients (~1-month post-stroke) with subcortical lesions and 8 with cortical lesions. The results showed that in the subcortical group, touch discrimination correlated negatively with the activation in the ipsilesional SI, SII, contralesional thalamus, and frontal attention regions; but in the cortical group, there was no significant correlated activity. However, this finding had some subtle differences from their later study (29). Five years later, they conducted a similar fMRI study to compare four stroke patients with subcortical lesions (thalamic/capsular) and seven with cortical lesions (the SI/SII) in the chronic phase of recovery and reported activation of ipsilesional supramarginal gyrus in stroke patients with thalamic or capsular lesions and no common activation in stroke patients with SI or SII lesions (29). Despite the small sample size, those studies provided novel insight into lesion-specific mechanisms of brain reorganization and potential therapeutic strategies to target specific brain regions, which warrant further investigation.

In addition, some imaging studies on the subject of recovery have compared stroke patients at an arbitrarily chosen time point (mainly at the chronic stage) with healthy controls to investigate the changes in neural activity and functional connectivity. However, those results were not sufficient to account for the possible time-dependent dynamics during the recovery, which called for more studies that would longitudinally investigate stroke patients in the process of recovery. For this purpose, Bannister et al. (32) conducted a longitudinal fMRI study to identify long-term changes in FC of the somatosensory network in 10 stroke patients with impaired touch sensation at 1- and 3-months by using seed-based analysis and touch sensation was evaluated by TDT. Compared to HC, 1-month post-stroke patients exhibited disruption of inter-hemispheric FC of homologous SI regions, whereas, at 6 months, there was some return of interhemispheric SI connectivity. This “return to more normal patterns” may be viewed as being associated with better recovery in the chronic stage (144, 145). Furthermore, correlation analysis was performed and great improvement in TDT scores was reported to be associated with significant FC changes within the contralesional hemisphere, i.e., great FC between contralesional SI and cerebellum at 1 month and great FC between contralesional thalamus and SII at 6 months. Those findings highlighted the important role of the contralesional hemisphere in post-stroke somatosensory performance and recovery, which was also purposed in the previous case reports (146, 147). Besides functional plasticity, there is a hypothesis that changes in brain morphology over time may also impact recovery. As we mentioned above, a longitudinal study conducted by Kessner et al. demonstrated different lesion-symptom patterns over time. They recruited 101 patients at the baseline, and finally, only 46 patients completed the entire study with all 3-time point measurements (at first 5 days and 3 months, 12 months follow-up) and used RASP to measure sensory deficits. Using VLSM, they found that a cluster of brain regions was significantly associated with lower RASP scores at baseline and 3-month post-stroke but no clear association between stroke lesion and sensory performances were observed at 12 months, which suggested that other factors beyond lesioned locations may account for somatosensory recovery. The brain atrophy rate from 3 to 12 months post-stroke was reported to be more than twice that of age-matched HC (148). There is some evidence that areas, such as the thalamus, hippocampus, and insula show greater atrophy after stroke (149–151). Yassi et al. (31) explored the long-term changes in the contralesional hemisphere in 15 acute middle cerebral artery territory ischemic stroke patients by observing volume change and FC within 1 week of onset and at 1 and 3 months. They have identified significant contralesional thalamic atrophy in the first 3 months and a positive correlation of atrophy degree with initial stroke severity. This result corroborated the finding that hypoperfusion in the thalamus could be observed after stroke in the acute and subacute stages, which accounts for a good or bad recovery (152–156). However, a recent retrospective cross-section study didn't identify a significant longitudinal relationship between total thalamic volume and time since a stroke in the chronic stages (157). This result was unexpected to some extent as previous studies have reported a negative correlation in the acute and subacute stages. All those findings raise the following possibility: structural thalamic adaption may be restricted to the acute or subacute stages and the changes in chronic stages (particularly after 3 months) may be too subtle to be detected using current techniques (157). In additional, Longitudinal changes in structural and functional features in stroke patients with somatosensory deficits were shown in Supplementary Table S1.

## Discussion

### Main Findings

Stroke is the most common cause of various degrees of functional impairments and neurological deficits, ranging from mild to severe. Somatosensory deficits after stroke are a common symptom caused by impairments of the central nervous systems. Previous studies utilizing neuroimaging tools have revealed detailed knowledge about a network of brain areas involved in the processing of somatosensory information. As mentioned in the first section, the whole process involves a functionally diverse set of brain regions, including the thalamus, SI, SII, PPC, insula, and cerebellum. Although there is a good description of a brain network implicated in the processing of somatosensory information, we still know less about the underlying mechanisms of somatosensory deficits and map plasticity during recovery. To summarize, this review has provided an up-to-date summary of the available data from MRI studies. Both structural MRI and fMRI studies have generated achievements in fundamental neuroscience research and clinical applications.

For structural MRI, the clinical applications of VLSM among stroke survivors have provided an important method for exploring potential structural abnormalities of neurologic somatosensory disorders. These studies generally suggest that stroke lesions occurring in the SI, SII, and IC could lead to somatosensory symptoms, but the one-to-one correspondence between a specific lesion pattern of brain regions and a certain somatosensory modality remains unclear. Moreover, the application of the structural brain connectome suggested that network disruption could explain the remote effects of a stroke lesion on additional variation in clinical outcomes. Recently, there is an apparent shift in the focus of fMRI. A series of ReHo and ALFF studies agreed on the notion that a positive correlation between somatosensory functions and cortical activation in stroke patients. Equally important was the finding that somatosensory deficits are a common symptom following lesions of the parietal lobe. Then, we also reviewed the state-of-the-art methods developed to analyze FC by using fMRI. A reduction of inter-hemispheric symmetry across the somatosensory network governing sensory perception could be observed in post-stroke somatosensory deficits. Some other functional imaging studies have revealed an increased FC of sensory-related regions in chronic stroke patients with sensory deficits.

Those results also emphasized the need to view the involvement of the neuroplasticity in somatosensory recovery after stroke, although the specific concept of the compensatory effect remains speculative. Current knowledge of post-stroke motor recovery and different patterns of neuroplasticity has been extensively identified (158, 159). However, little is known about sensory recovery. Limited studies have suggested that the brain could reorganize to optimize somatosensory recovery and the process may be influenced by the lesion location and course of stroke (15, 28, 160, 161). Thus, recovery varies among individuals, resulting in the heterogeneity of stroke outcomes. It is acknowledged that gray matter atrophy co-exists with brain plasticity presenting with structural and functional remolding in specific regions (162). The substantial recovery of subcortical lesions, particularly thalamic stroke, maybe due to the involvement of the thalamo-cortical circuit and sensori-motor networks. In addition, activation patterns of corresponding brain areas may differ between cortical lesions and subcortical lesions. We believe that a better understanding of the underlying neurological mechanisms could contribute to the development of novel therapeutic strategies that will promote repair and alleviate somatosensory deficits.

### Limitations and Challenges

Although studies obtained by this review provide potential insights into the neural substrate of post-stroke somatosensory deficits, there are many conflicting results obtained by extensive studies. Those controversies may probably be attributed to the study design, suggesting current research has some shortcomings that are yet to be solved. Therefore, several limitations and challenges will be listed in the following paragraphs.

In contrast to a large number of studies that applied those approaches in the field of motor symptoms and motor recovery after stroke, a relatively small number of studies have investigated somatosensory deficits after stroke. There are several possibilities to interpret this apparent situation. One important reason is that the clinical need for motor recovery is greater than that for sensory recovery, though intact sensory function could promote motor recovery (163). Another possibility is the difficulties in recruiting patients with pure sensory stroke. The presence of somatosensory deficits, combined with motor dysfunction, makes interpretation difficult. The second drawback of this review is the small sample size of original MRI studies in this field of research. Many included studies have a limitation of a small number of patients because the costs of MRI were prohibitive for larger studies. Recent evidence indicates that limited sample sizes may yield mixed results in MRI studies, although there is no consensus on the sample size calculation (164). The small samples may lack enough statistical power. Thus, it is not sufficient to draw firm conclusions as the evidence is mixed.

Beyond the reasons discussed above, we speculate that different results obtained by different studies may also depend on the heterogeneity in clinical measurement of somatosensory deficits, which is the most complicated part of the neurologic investigation. Compared to assessments for motor dysfunction, somatosensory assessments in current clinical practice are less reliable and reproducible due to the lack of consensus on sensory paradigms (165). Obviously, different studies adopted different scales to measure somatosensory performances, resulting in different results. Thus, to reduce the clinical heterogeneity of somatosensory outcomes, it's imperative to establish consensus on the scales for scientific research. Furthermore, the complexities of cortical interactions and the multitude of sensory modalities also require doctors or therapists, who are equipped with a great amount of knowledge and experience in neurology to make a diagnosis. Equally important is the cooperation and compliance of the patients and they should try to understand that this procedure is the least objective part of a neurologic examination. Taken together, the complexity of sensory assessments may contribute to the paucity of quality research in this field.

### Future Perspectives

There is considerable variability among the currently available findings. Those discrepancies may be attributable to the small sample size of studies, heterogeneity in the clinical phenotypes, and differences in the clinical assessments or analytic methods. The assumptions formulated by included studies can also provide several suggestions for future directions of the research in this field.

Firstly, it should be noted that the thalamus didn't show up in most VLSM studies, despite its importance in somatosensory processing. This could be possibly due to the small number of patients who had lesions in the thalamus in those studies. Thus, future research should address the MRI scanning and somatosensory assessment with respect to lesion-symptom associations in patients with thalamic stroke. Besides, whether a specific lesion pattern may account for a certain somatosensory impairment remains elusive. More studies using VLSM, or other analytical methods should focus on the one-to-one correspondence between a specific lesion pattern of brain regions and a certain somatosensory modality. We think this may help to explain the variability in deficits across patients. And then, many FC studies have suggested that a focal stroke lesion affects not only the peri-lesional region but also the pathway or network to which it belongs. For example, the lesions in the thalamo-cortical circuit were considered vulnerable to cause somatosensory deficits, and increased FC within this circuit could be observed in the process of somatosensory recovery (19, 27). It is acknowledged that the thalamus and some cortical regions (e.g., the temporal gyrus and the occipital gyrus) could share and communicate sensory information and are linked on an anatomic basis. There is a hypothesis that somatosensory deficits can be regained due to the compensation of the thalamo-cortical circuit. But this hypothesis requires further experiments to confirm. Similarly, one previous fMRI study emphasized the importance of the sensori-motor network in somatosensory recovery (27). Accumulating evidence suggested that intact sensory information could enhance motor cortex excitability. Although the exact mechanisms of sensori-motor interactions remain unknown, there is increasing evidence that sensori-motor disconnection could cause sensory or motor dysfunction (11, 142). More future work should focus on the interaction between sensory and motor systems: how does a simple loss of one system cause functional impairments in the other one; how does sensori-motor execute its function in the process of somatosensory recovery. Moreover, we speculate that the stroke lesions following subcortical or cortical regions may have different activation patterns during recovery of somatosensory and we think it is worthy of further examination to address this issue. This could be executed through more restrictive inclusion criteria in future research, for example, the lesion location should be restricted to a specific subcortical area. More restrictive inclusion criteria could lead to more homogenous groups or rigorous experimental control resulting in higher sensitivity. Finally, all those speculations need to be confirmed by more high-qualified studies with larger sample sizes, especially longitudinal studies. It is acknowledged that longitudinal studies using MRI techniques could assess structural and functional changes in the brain over time in order to investigate the “return to normal pattern.” Thus, longitudinal studies with a large sample size that span the acute to chronic phases are required to determine the underlying possible compensatory mechanisms of somatosensory deficits and represent a future target for therapy.

Whilst limited studies hamper our understanding of the pathogenesis of post-stroke somatosensory deficits, some methodological issues in the existing MRI studies could contribute to the understanding of the progress in the application of MRI techniques in this field. In this paper, we summarized an updated account of advances in MRI. We hope that this review will equip new researchers with a more complete understanding of advanced MRI techniques and assist them in launching more comprehensive studies. As seen from the studies presented here, many studies only used one single MRI modality, making it difficult to directly investigate the synchronous structural and functional changes in the same samples. Considering that different MRI modalities provide different aspects of neural alterations, it is anticipated that methods combining functional and structural data to find out the neuroimaging biomarkers will be of value. With regard to fMRI, the number of studies that applied the ReHo and ALFF was relatively small. We hope that those two methods will be used far more widely for analyzing the activations of specific brain regions and revealing the functional segregation. In comparison, FC has contributed more to the field of post-stroke somatosensory deficits. Also, how to choose an appropriate method to analyze FC has been a long-time discussion for novice researchers (76, 166, 167). The preference and selection of a method are dependent on the scenario and study design. As we mentioned previously, most studies used seed-based methods, whilst only one study has tried to apply the graph theory to examine the whole brain. Undoubtedly, selections of seeds in different studies were identical, which may add to selection bias, and then the results may be different. Graph theory can help in modeling the brain as a complex network represented graphically, thereby solving the problem caused by seed-based analysis. Thus, there is a great need for more research using the graph theory to improve our understanding at the whole-brain level. Furthermore, the hypothesis on lateralized processing of sensation is also worth noting. We have noticed that many studies didn't take hemisphere-specific information into consideration. In many conditions, if the lesion locations were disturbed in both hemispheres, data would be flipped to one side so lesions would be represented within a single hemisphere (19, 20, 22, 26, 28, 29, 32). This “flipping” method seems to make it simpler and more convenient to analyze the data, but the differences in FC associated with the side of the lesion were therefore hidden (24). Despite the effects of flipping have not reached a consensus, we still think it is better not to flip the MRI data before pre-processing, since patients with right hemisphere stroke may be more likely to have somatosensory deficits (19). Therefore, future studies using a multimodal MRI approach, unbiased whole-brain analytical methods, and taking into account the lateralization when examining somatosensory deficits will be required to elucidate the exact mechanisms.

Although MRI is considered a relatively mature method, ongoing improvements in analytical strategies and hardware continue to improve our knowledge of the mechanisms underpinning brain function and disease (100). Other well-developed methodological approaches, such as TMS, diffusion tensor imaging (DTI), Arterial Spin Labeling (ASL), magnetic sensitivity weighted imaging (SWI), quantitative sensitivity magnetization (QSM), and magnetic resonance spectroscopy (MRS) should be given due importance and incorporated into future studies. Besides analytic methods referred to above, other methods can also be applied to analyze the MRI data. For example, another two methods to analyze rs-fMRI data are known as clustering algorithms and multivariate pattern classification (168). The former could group a collection of voxels on the basis of similarities, and the latter could be used to predict individual brain maturity. Moreover, with the rapid advancement of artificial intelligence, machine learning, and especially deep learning technologies are more and more widely used in MRI studies (169–174). Such new and valuable tools may spur radioligand development for extending our knowledge of the pathogenesis of somatosensory deficits and informing treatment strategies.

## Conclusion

Somatosensory deficits may occur in 50–80% post-stroke patients and thereby negatively affect the quality of life. It has a strong and negative impact on brain performance and life quality. The intact somatosensory system is crucial for recovering motor behavior and improving other body functions after a stroke. Thus, a better understanding of the underlying neurological pathway of post-stroke somatosensory deficits may contribute to the development of targeted therapies. The past two decades have greatly deepened our knowledge of MRI. Various strategies have been used to assess neural changes associated with functional deficits and stroke recovery. However, a limited number of papers regarding the clinical application of MRI techniques among patients with post-stroke somatosensory deficits have been published. MRI techniques, mainly including structural MRI and fMRI, allow changes in brain structure and activity after stroke to be identified. The information provided in the aforementioned sections indicates that advanced MRI techniques have been used in clinical studies. The results from those preliminary studies have demonstrated that the parietal cortex, as the anatomical sites of the SI and SII, contributes to somatosensory symptoms. In addition, some subcortical regions, including the IC and thalamus are also involved. Moreover, a reduction of inter-hemispheric symmetry across the sensory-related network and increased FC of sensory-related regions could be observed simultaneously, revealing a phenomenon that gray matter atrophy co-exists with brain plasticity. Nonetheless, many uncertainties and controversies still exist. The clinical variability of study subjects and differences in clinical measurements and analytical strategies may contribute to discrepant findings in these studies. Further work is needed to determine the generalizability of somatosensory deficits across stroke patients with different lesion locations and characteristics.

## Data Availability Statement

The original contributions presented in the study are included in the article/Supplementary Material, further inquiries can be directed to the corresponding author/s.

## Author Contributions

QL conceived and wrote the review article. JZ and YP independently conducted the search and screened the titles, abstracts, and full texts of the papers. XL and LM revised the article. JP and LS analyzed the data and drew the figure and table. YZ and XC gave final approval for publication. All authors contributed to the article and approved the submitted version.

## Funding

This work was supported by the National Natural Science Foundation of China, Grant Number: 81904285.

## Conflict of Interest

The authors declare that the research was conducted in the absence of any commercial or financial relationships that could be construed as a potential conflict of interest.

## Publisher's Note

All claims expressed in this article are solely those of the authors and do not necessarily represent those of their affiliated organizations, or those of the publisher, the editors and the reviewers. Any product that may be evaluated in this article, or claim that may be made by its manufacturer, is not guaranteed or endorsed by the publisher.
