# Supplementary material for: Somatosensory Deficits After Stroke: Insights From MRI Studies
Source: Front Neurol. 2022 Jul 12;13:891283. doi: 10.3389/fneur.2022.891283 (PMC9328992; doi:10.3389/fneur.2022.891283)
Supplement: Supplementary file 1 [file Table_1.pdf]

Table S1 longitudinal changes in structural and functional features in stroke patients with somatosensory deficits

		Time point			Main findings		
Reference	Subjects	1-stage	2-stage	3-stage	1-stage	2-stage	3-stage
Bannister et al., 2015(1)	10SP/10HC	1-month	6-month	/	Greater improvement of TDT scores was associated with greater FC between contralesional SI and contralesional cerebellum and hippocampus	Greater improvement of TDT scores was associated with greater FC between contralesional SII and IPC and contralesional thalamus and cerebellum.	/
kessner et al,2019(2)	101IS	within 5 days of onset	3-month	12-month	Significant associations between lower RASP scores and lesioned brain tissue were found in the S1, S2, IC and frontoparietal subcortical areas.	Only the S1 and S2 showed significant involvement with lower RASP scores.	no statistical significance was found between lesion sites and RASP scores.
Yassi et al,2015(3)	15IS/7HC	within 7 days of onset	1-month	3-month	The mean volume of the contralesional thalamus was lower than HC but not statistically signific ant.	Contralesional thalamic volume was significant reduced compared to baseline.	The degree of atrophy was significant reduced compared to baseline and correlated with initial stroke severity; a significant linear reduction in FC between the contralesional thalamus and the rest of the brain.

Abbreviation: FC, functional connectivity; HC, health control; HOFC, high-order functional connectivity; HS, hemorrhagic stroke; IS, ischemic stroke; MRI, magnetic resonance imaging; RASP, Rivermead Assessment of Somatosensory Performance; SP, stroke patients; TDT, Tactile Discrimination Test.

1. Bannister LC, Crewther SG, Gavrilescu M, Carey LM. Improvement in Touch Sensation after Stroke is Associated with Resting Functional Connectivity Changes. *Front Neurol* (2015) 6:165. doi: 10.3389/fneur.2015.00165

2. Kessner SS, Schlemm E, Cheng B, Bingel U, Fiehler J, Gerloff C, Thomalla G. Somatosensory Deficits After Ischemic Stroke Time Course and Association With Infarct Location. *Stroke* (2019) 50:1116–1123. doi: 10.1161/strokeaha.118.023750

3. Yassi N, Malpas CB, Campbell BCV, Moffat B, Steward C, Parsons MW, Desmond PM, Donnan GA, Davis SM, Bivard A. Contralesional Thalamic Surface Atrophy and Functional Disconnection 3 Months after Ischemic Stroke. *Cerebrovasc Dis* (2015) 39:232–241. doi: 10.1159/000381105
